# Design and analysis of photovoltaic/wind operations at MPPT for hydrogen production using a PEM electrolyzer: Towards innovations in green technology

**DOI:** 10.1371/journal.pone.0287772

**Published:** 2023-07-20

**Authors:** Mohamed Awad, Mohamed Metwally Mahmoud, Z. M. S. Elbarbary, Loai Mohamed Ali, Shazly Nasser Fahmy, Ahmed I. Omar

**Affiliations:** 1 Department of Electrical Power & Machines Engineering, Faculty of Engineering, Cairo University, Giza City, Giza, Egypt; 2 Electrical Engineering Department, Faculty of Energy Engineering, Aswan University, Aswan, Egypt; 3 Electrical Engineering Department, College of Engineering, King Khalid University, Abha, Saudi Arabia; 4 Electrical Power and Machines Engineering Department, The Higher Institute of Engineering at El-Shorouk City, El-Shorouk Academy, Cairo, Egypt; J.C. Bose University of Science and Technology, YMCA, INDIA, INDIA

## Abstract

In recent times, renewable energy systems (RESs) such as Photovoltaic (PV) and wind turbine (WT) are being employed to produce hydrogen. This paper aims to compare the efficiency and performance of PV and WT as sources of RESs to power polymer electrolyte membrane electrolyzer (PEMEL) under different conditions. The study assessed the input/output power of PV and WT, the efficiency of the MPPT controller, the calculation of the green hydrogen production rate, and the efficiency of each system separately. The study analyzed variable irradiance from 600 to 1000 W/m^2^ for a PV system and a fixed temperature of 25°C, while for the WT system, it considered variable wind speed from 10 to 14 m/s and zero fixed pitch angle. The study demonstrated that the applied controllers were effective, fast, low computational, and highly accurate. The obtained results showed that WT produces twice the PEMEL capacity, while the PV system is designed to be equal to the PEMEL capacity. The study serves as a reference for designing PV or WT to feed an electrolyzer. The MATLAB program validated the proposed configurations with their control schemes.

## Introduction

Hydrogen is a technique for decarbonizing solutions because it burns without releasing CO_2_. Although it is not the main energy source like oil, coal, or gas, it may be utilized as a transporter for energy. With most renewable energy sources (RESs), energy storage is necessary [1]. Hydrogen can be produced from RESs, calling it green hydrogen. Green hydrogen has a considerably larger potential than fossil fuels because it is related to Photovoltaic (PV) and wind turbine (WT), which greatly exceeds global energy consumption today and, in the future [1]. PV is a new form of energy that is created by relying on sunshine, whereas WT is a RES that is dependent on wind speed to operate. PV or WT powers an electrolyzer to produce hydrogen. A polymer electrolyte membrane electrolyzer (PEMEL) is one type of electrolyzer that uses electricity to break water into hydrogen and oxygen.

Most of the literature works focused on two ways, the first trend in green hydrogen which is powered by a renewable energy grid-connected system, and the second direction in green hydrogen which is powered by RES in an off-grid system. Mokhtara et al. optimized the sizing of an on-grid hybrid RES that contains PV, electrolyzer, fuel cell (FC), and storage tank (ST) for a university building in the south of Algeria, using a particle swarm optimization (PSO) method and Homer program [2]. The integration of a hydrogen generator with a WT including well power characteristics was investigated by Kotowicz et al. [3]. Alkaline electrolysis (AEL) and PEMEL dynamic behavior for grid-connected PV and WT input data sets from the region of northwest Germany were examined by Schnuelle et al. [4]. Yang et al. offered a detailed strategy for figuring out the capacity construction and power reallocation of a PV-hydrogen on-grid system [5]. Power management controls were created by Dahbi et al. to reduce extra energy in a PV on-grid system while producing green hydrogen [6]. Yildirim, created a smart controller based on the WT, PV, FC, electrolyzer, battery energy storage systems (BESS), and loads to efficiently execute load frequency regulation of an island’s FC microgrid [7, 8]. Atawi et al. proposed a controlled microgrid that includes WT/FC units that supply both dynamic and static loads [8]. The design and control techniques of a PV/FC microgrid hybrid system were optimized by Ghenai et al. to handle the electrical demands of 150 homes in Sharjah, United Arab Emirates [9]. An autonomous clean energy microgrid using a regenerative hydrogen fuel cell (RHFC) as a backup generator for up to 10 days was proposed by Jansen et al. [10].

In an off-grid application, Attemene et al. recommended the ideal configuration of a system made up of a WT/ PEMFC/AEL/battery/super-capacitor bank [11]. The electrical dynamics of the PV/battery/FC/electrolyzer were modeled and experimentally validated by Badoud et al. [12]. Hasan and Dincer developed a RES-based integrated system that consists of a WT/PV/electrolyzer/ ammonia synthesis system [13]. Douak and Settou suggested a model of an effective hybrid system PV/WT/ FC that covers the needs of a typical off-grid home in the Adrar region of Algeria [14].

In various studies on the performance of RESs in powering PEMELs, researchers have examined the effectiveness of different systems. Albarghot and Rolland compared the simulation and experimental data of a PEMEL powered by a horizontal WT [15], while Akyuz et al. used MATLAB Simulink to examine a hybrid system that includes WT/PV/battery/PEMEL [16]. Albarghot and Rolland contrasted the outcomes of a PV-powered PEMEL simulation and experiment [17]. Zhang et al. investigated the efficiency of a 3 kW PV and 3 kW PEMEL system in different surroundings [18]. Table 1 summarizes the literature on green hydrogen-powered by RESs.

**Table 1:** A review of the literature based on green hydrogen-powered by RESs.

Ref.	Objectives	Program/device used/ method	Important conclusions
[6]	Design and modeling of a hybrid system (PV/grid) that powers an electrolyzer that generates clean hydrogen and powered a DC load.	MATLAB/ power management control	Development of the maximization of the PV modules’ power, securing the regular supply of the load, and optimizing the creation of hydrogen.
[14]	Development of a model of an effective hybrid system made up of PV, WT, and FC components for the Adrar site.	FORTRAN language	According to this study, the PV/Wind/FC system is the best choice for the Adrar site.
[9]	Designed and controlled techniques of a PV/FC microgrid hybrid system to handle the electrical demands of 150 homes in Sharjah.	Power management system (PMS)	The findings demonstrate that the PV/FC/Electrolyzer/Inverter system is capable of supplying the daily electrical needs of the 150 homes in the residential community that was chosen.
[11]	Determining the best configuration for an off-grid application involving a WT, a PEMFC, an AEL, a battery, and a supercapacitor banking.	Non-dominated Sorting Genetic II (NSGA-II) algorithm	In regions with significant wind potential, the suggested structure would be economical. However, the incorporation of a renewable source like PV should be looked into in places with poor wind potential.
[5]	Investigating a general approach to deciding the capacity configuration and power reallocation for a grid-connected PV system integrated hydrogen generation system.	Monitoring inverter output and experimental data.	The grid connection configuration and hydrogen production status affect the best reallocation of PV-hydrogen power; and a large-scale PV system integrated with a hydrogen system can improve power utilization, economic benefits, and emissions
[8]	Controlling microgrid that includes WT/FC units that supply both dynamic and static loads.	Control algorithm using the Mine Blast Algorithm (MBA) and MATLAB	As a result, when the wind speed is low, the FC feeds the loads like a slave.
[12]	Modeled, and experimentally validated the electrical dynamics of the PV/Battery/FC/Electrolyzer.	Power management algorithm and experimental study.	The experimental findings demonstrate that the control approach employed successfully controls the electrolyzer and FC working patterns while saving the battery bank against excessive use.
[4]	Analyzed AEL and PEMEL dynamic behavior for on-grid PV and WT input data sets from the region of northwest Germany.	key performance indicators (KPI) like costs and efficiencies.	From an economic standpoint, AEL technology is preferred for electrolyzer functioning with a direct renewable electricity source.
[15]	Compared the simulation of a PEMEL powered by a horizontal WT and the experimental data of it.	MATLAB/ experimental set-up.	The results from the simulation low higher than the experimental setup.
[16]	Analyzed the performance of a hybrid system that includes WT/PV/Battery/PEMEL.	MATLAB software.	The energy efficiency of the PEMEL at 35–75°C was found to be 64–70%.
[3]	Analyzing the integration of a hydrogen generator with a WT system including sufficient power characteristics	Laboratory examination.	The hydrogen generator-rated power is equal to the WT-rated power, and it is dependent on the wind speed and the amount of power from the WT which is available.
[17]	Comparing the outcomes between a PV-powered PEMEL simulation and its experiment.	MATLAB program and experimental test.	The maximum power of the PV panel is influenced by the weather. The hydrogen production rate from the simulation is higher than the results from the experiments test.
[2]	Development of the sizing of an on-grid HRES that contains PV, electrolyzer, FC, and ST for a university building in the south of Algeria.	PSO method/ Homer program	The ideal PV system for the chosen structure is the grid. The grid-connected PV-H_2_ is the optimum option for the building and transportation sector.
Proposed	Design and analysis of PV and WT systems to produce hydrogen using a PEMEL and MPPT controller.	MATLAB	The Power of WT to feed PEMEL is to be approximately two times PEMEL capacity. In addition, the PV system is designed to be equal to PEMEL capacity.

This paper introduces a PV and WT system to produce green hydrogen using PEMEL, a buck converter to reduce the outage voltage to the operation voltage of PEMEL, and applying P&O-MPPT to get the maximum power of the system. Results obtained the efficiency and economical of the PV and WT systems to produce hydrogen.

The main contributions of this study can be categorized as follows:

- Design and implementation of PV and WT system-powered PEMEL as a stand-alone system.
- Implementing P&O MPPT to each system to get maximum power from PV and WT systems using low incremental to get fast speed response from the systems.
- It measured the input, output, and efficiency of the MPPT controller under the conditions of variance irradiance level from 600 to 1000 W/*m*^2^ and 25°C fixed temperature for a stand-alone PV system. Furthermore, it measured the input, output, and efficiency of the MPPT controller under the variable wind speed from 10 to 14 m/s and zero fixed pitch angle for the WT system.
- Measured the hydrogen production rate, and characteristics of voltage and current for each system. Finally, making the comparison between PV and WT systems results feed the same PEMEL and shows the efficiencies, and differences, and makes a comparison between PV system output and other work in this field.
- This paper can be a guide to designing PV or WT that will feed an electrolyzer in practical life.

The rest of the paper is organized as follows: Section 2 presents the design and methodology for the main components (PEMEL, PV, WT, and Buck converter with MPPT function). In Section 3, results and discussion are provided to compare the PV system and WT system with the PEMEL. In Section 4, conclusions about green hydrogen using the PV and WT systems and the future challenges for green hydrogen are presented.

## Design and methodology

The system consists of these elements which are PEMEL, PV or WT system, buck converter, and the MPPT function.

### Polymer electrolyte membrane electrolyzer

The electrolyzer converts electrical energy from a renewable energy source (PV or WT) into hydrogen and oxygen, and then the hydrogen is stored in a tank [19]. The PEMEL is made up of a solid polymer electrolyte (SPE) which is responsible for transferring protons from the anode to the cathode and separating the generated gas at the anode and cathode. In addition, providing electrical insulation between the two electrodes while operating as a reactant shield against gas crossover [20]. PEMEL is better than other types of electrolyzers because it can start quickly, which is important in renewable energy systems due to variations in solar irradiances and wind speeds [21].

The following are the chemical processes of PEM water electrolysis:

$$Anode:\;2H_{2}O\to O_{2}+{4H}^{+}\;{+4e}^{-}$$ (1)

$$Cathode:\;{4H}^{+}{+4e}^{-}\to 2H_{2}$$ (2)

$$Global:\;2H_{2}O\to 2H_{2}+O_{2}$$ (3)

**Fig 1:**
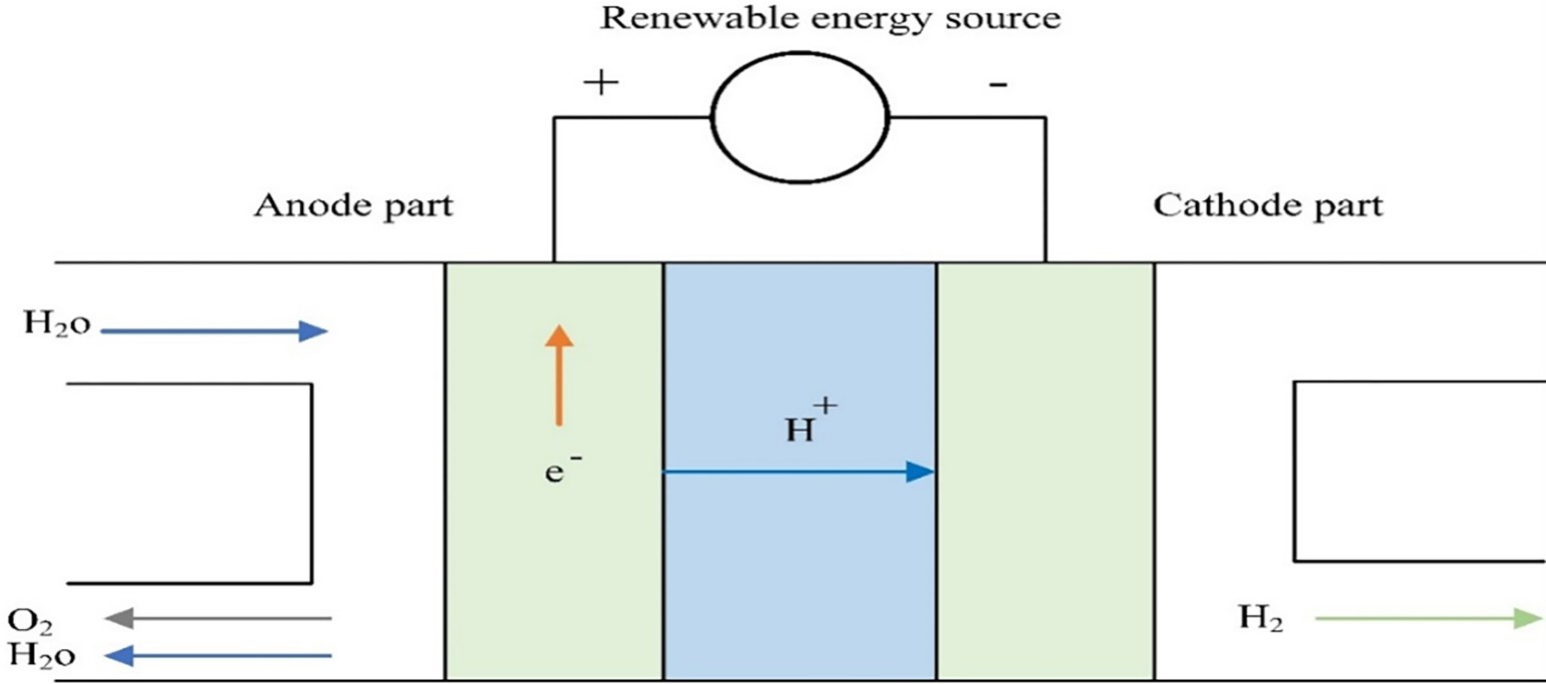
Operation concept of the PEMEL.

Water particles react at the anode to create oxygen and positively charged protons as a result of the reactions. Protons then travel through the SPE on their way to the cathode, where they interact with electrons to form hydrogen. Fig 1 obtains the PEMEL operation concept [20].

The flow rate of hydrogen production by the PEMEL is calculated according to the following equation [22]:

$$F_{H2}=\frac{N\;I}{n\;F}\;\eta_{F}$$ (4)

where F_H2_ represents the electrolyzer hydrogen flow rate in (mol/s). N denotes the electrolyzer cell number. η_F_ denotes Faraday’s efficiency. *I* referred to the electrolyzer operating current. F is Faraday’s constant (96485 C/mol). N represents the number of electron exchanges (n = 2) [22, 23]. Faraday’s efficiency can be calculated as follows [24]:

$$\eta_{F}=96.5\;\exp(\frac{0.09}{i_{el}}-\frac{75.5}{i_{el}^{2}})$$ (5)

In actual life to calculate the flow rate of hydrogen production in (mol/s), you should put electrolyzer efficiency η_el_ in your consideration which is put in Eq (6) as follows:

$${\;F}_{H2}=\frac{N\;I}{n\;F}\;\eta_{F}\;\eta_{el}$$ (6)

According to International Renewable Energy Agency (IRENA), the average PEM electrolyzer efficiency η_el_ currently equals 60% [25]. The rate of hydrogen generation is given in Nm^3^/h as follows [23]:

$$Q=F_{H2\;}\times 3600\times 0.022414$$ (7)

where Q is the hydrogen flow rate in N*m*^3^/h. The higher heating value (HHV) of hydrogen is the heat of formation (enthalpy); the least amount of energy required to split water into hydrogen and oxygen. The HHV is defined as the amount of energy released by fuel combustion starting at 25°C and allowing the products to cool to 25°C after combustion [26]. The PEMEL used for analysis is from National Renewable Energy Laboratory (NREL) project, named (PEM E-130 stack). Table 2 shows the parameters of the PEM E-130 stack [26]:

**Table 2:** PEM E-130 stack parameters [26].

Item	Value
Specification	PEM E-130 stack
No. of series Cells	20 cells
Voltage per one cell	From 1.5 to 2.2 V
Operating current of the stack	From 0 to 200 A (135 A rated)
Operating voltage of the stack	43 V
Calorific value for hydrogen at HHV	286000 J/ml
Faraday’s efficiency	96.165%

### PV technology

**Fig 2:**
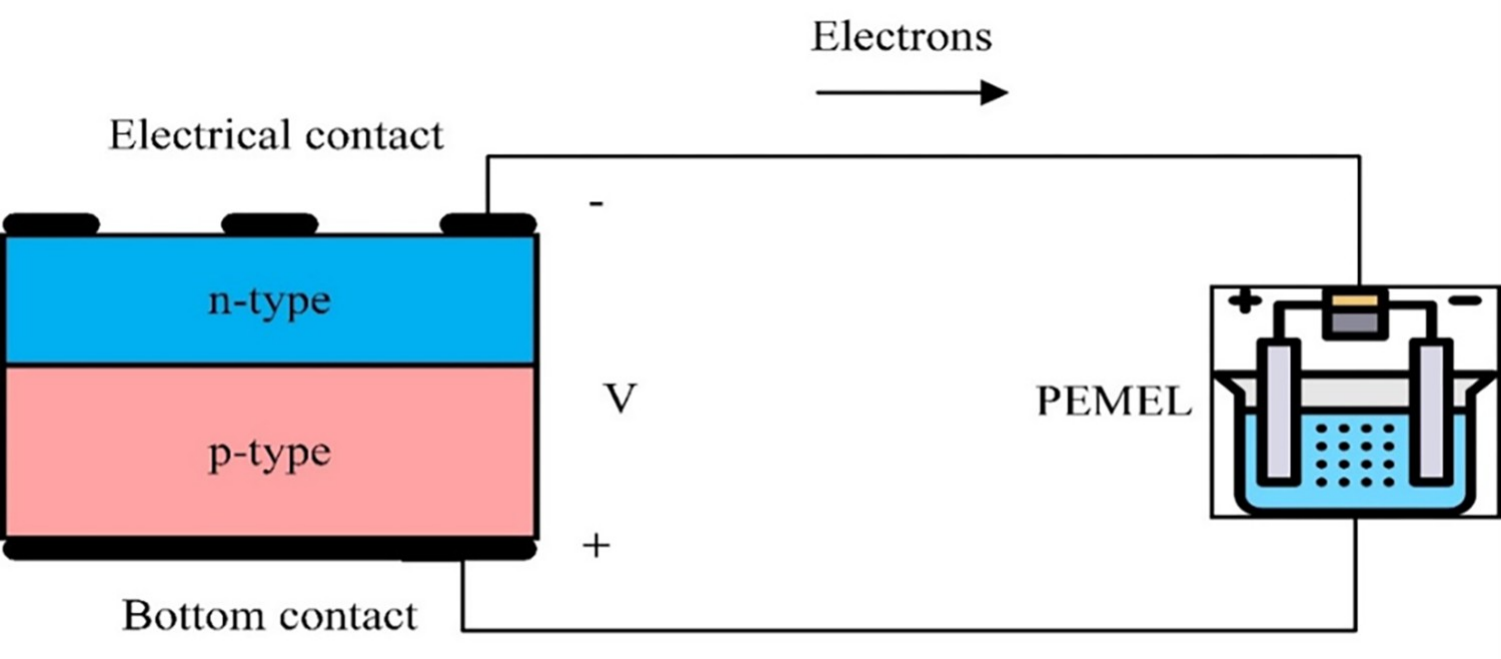
The principal operation of the solar cell.

PV panels use sunlight as a source of energy to generate direct-current electricity. Solar cells consist of two parts: n-type (negative part) and p-type (positive part). Although it is not the primary mechanism for endowing directionality to carriers in the majority of photovoltaic devices, elementary treatments of photovoltaics emphasize the importance of this field in separating charge carriers [27]. In the entire volume of the p-n junction, excited electrons convert the semiconductor’s valence band to the conduction band when absorbing light [27]. Mobile electrons migrate across the junction in an n-type material through diffusion. In addition, mobile holes in the p-type material move oppositely across the junction due to diffusion. When the sun shines on the solar cells, electrons flow from the n-type into the connecting wire, via the load, and back to the p-type, as shown in Fig 2 [28].

A PV array contains some modules in series and parallel as required for the load system. *I*_*m*_, *V*_*m*_, and *P*_*m*_ are the maximum current, maximum voltage, and maximum power of the PV array, correspondingly [29].


$$P_{m}=V_{m}I_{m}$$ (8)

**Fig 3:**
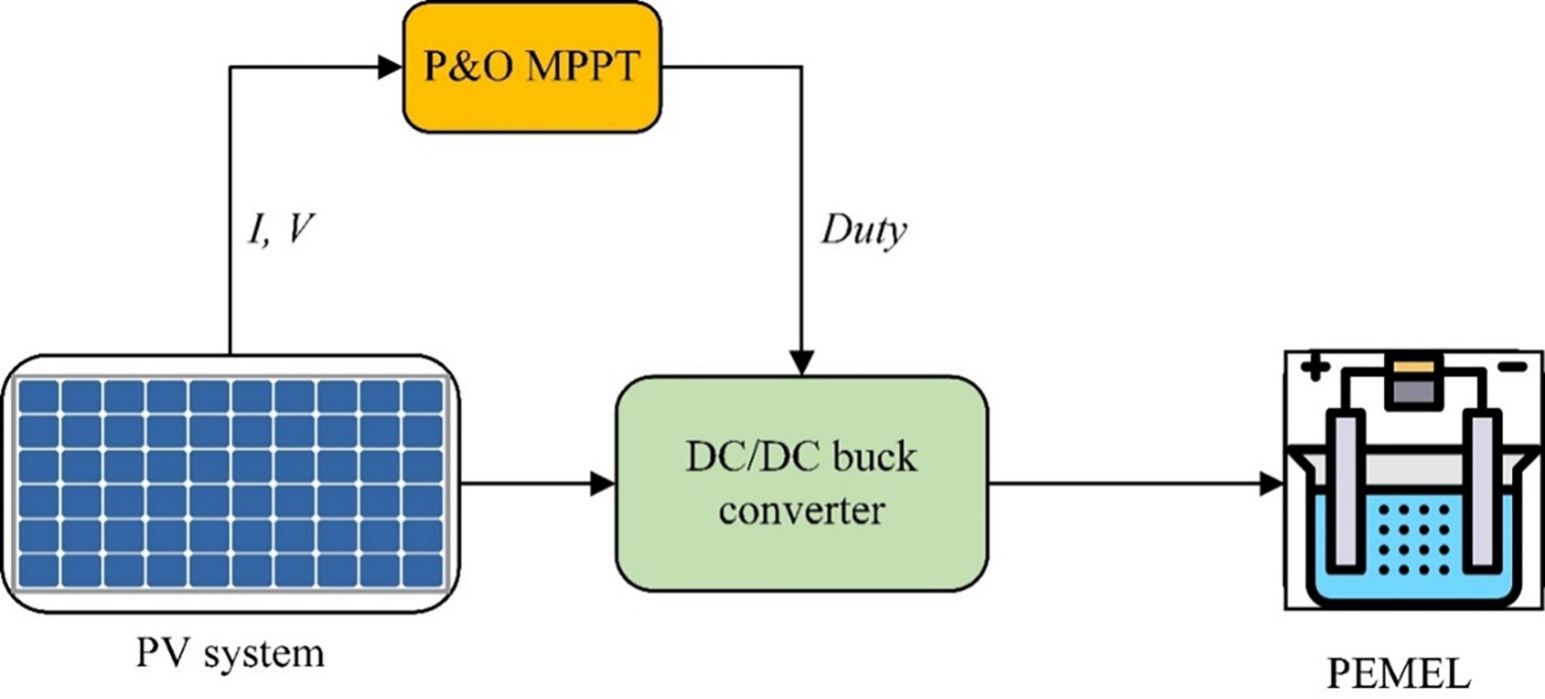
A diagram of the PV system with PEMEL.

Irradiance is higher meaning the higher power you can get from the PV system [30]. A temperature of 25°C or less is preferable to the PV system [30]. This model suggests using the PV array to power the PEMEL through the buck converter to step down voltage. Moreover, applying the MPPT technique to gain maximum power from the PV system, as shown in Fig 3 [31].

PV system efficiency can be calculated as follows [32]:

$$\eta_{PV\;system}=\frac{P_{output}}{G\;A_{PV}}$$ (9)

where G is solar intensity (1000 W/m^2^), A_pv_ is PV Modules area, and P_output_ is the output of the PV system (input power to electrolyzer). Overall efficiency for a PV system-powered PEMEL is calculated according to [32].

$$\eta_{PV+Electrolyzer}=\frac{F_{H2\;}E}{G\;A_{PV}}$$ (10)

where E is the Calorific value for hydrogen, in J/ml. The PV system parameters for simulation in MATLAB are as in Table 3 [33].

**Table 3:** PV system model parameters [33].

**PV module specifications**	
**Item**	**Value**
*P*_*m*_	350 W
*V*_*m*_	38.5 V
*I*_*m*_	9.09 A
*A*	(1.984 ×0.998) = 1.98 *m*^2^
**PV array specifications**	
No. PV Modules in series	2 Modules
No. of strings	10 Modules
Total power of PV system	7000 W
Input irradiance (Fixed)	1000 W/*m*^2^
**Buck converter specifications**	
Capacitor (*C*)	5.309e-3 F
Inductance (*L*)	1.16634e-5 H
*V*_*ripple*_	1% of *V*_*out*_ (4.3 V)
Load resistance (PEM electrolyzer)	0.26414 Ω
Series resistance to load	0.09 Ω
MPPT type	P&O MPPT

### Wind Turbine (WT) technology

The kinetic energy inside a WT is converted to mechanical energy, which is ultimately converted to electrical power by a generator [34]. The WT is linked to the PEMEL through a permanent magnet synchronous generator (PMSG), a three-phase rectifier, and a DC/DC buck converter [35, 36].

**Fig 4:**
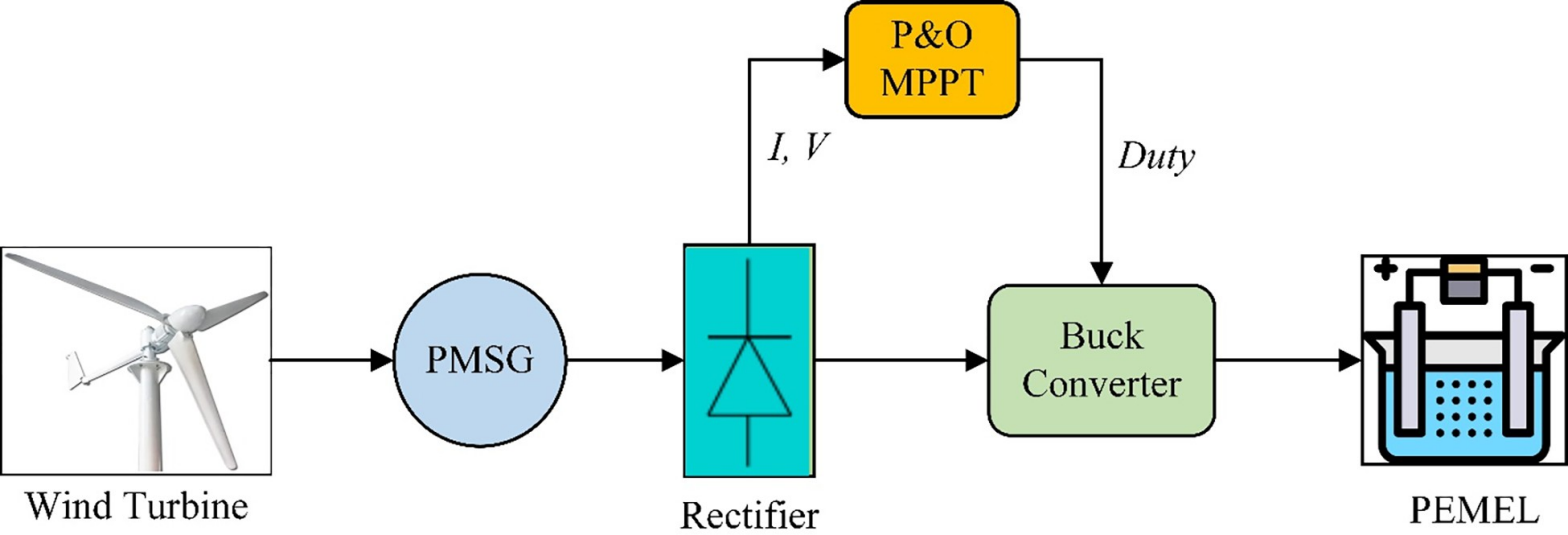
A diagram of the WT system with PEM electrolyzer.

PMSG is commonly utilized in stand-alone compact wind turbines due to its high efficiency and minimum maintenance and a three-phase AC voltage at the input is converted to a DC voltage at the output using a three-phase rectifier [37]. The MPPT controller is used to achieve the maximum output of the WT system as shown in Fig 4 [38].

This equation gives the mechanical power, *P*_*m*_, captured by the turbine [34, 35, 37]:

$$P_{m}=0.5\;\rho \;A\;V^{3}\;C_{p}(\beta ,\lambda )$$ (11)

where *C*_*p*_ refers to the power coefficient, *β* indicates the blade pitch angle, λ is the tip speed ratio (TSR), ρ represents the air density, *A* is swept area, and *V* is the wind speed [37].

The higher height of the tower means getting a high speed of the wind and this lead to getting more power from the WT system [39]. Offshore WT gives a higher speed than the onshore WT reflecting in getting more power also from the WT system [40]. The higher length of the diameter of the blades of WT the more power output [41]. The location of the WT system is the most factor that affecting in all parameters and outputs of the WT system [42].

The PMSG is simulated in the *dq* reference frame. The armature has induced a voltage on both the *d* and *q* axis. DC voltage and current are used to power the generator. The next equations determine the current on the *d* and *q* axes [37]:

$$\frac{{di}_{sd}}{dt}=-\frac{R_{sa}}{L_{sd}}{\;i}_{sd}+\frac{L_{sq}}{L_{sd\;}}\omega_{s\;}{\;i}_{sq}+\frac{1}{L_{sd\;}}u_{sd\;}$$ (12)

$$\frac{{di}_{sq}}{dt}=-\frac{R_{sa}}{L_{sq}}{\;i}_{sq\;}-\omega_{s\;}(\frac{L_{sd}}{L_{sq\;}}{\;i}_{sd}+\frac{1}{L_{sd\;}}\psi_{p})+\frac{1}{L_{sq\;}}u_{sq\;}$$ (13)

Eq 14 gives the electromagnetic torque (T_e_) delivered from the PMSG rotor [37]:

$$T_{e}=1.5\left(\frac{P}{2}\right)\;[\psi_{p}\;{\;i}_{sq}+{\;i}_{sd}\;{\;i}_{sq}(L_{sd}-L_{sq\;})]$$ (14)

The currents and voltages of the *d* and *q* axes are represented by *i*_*sd*_, *i*_*sq*_, *u*_*sd*_, and *u*_*sq*_, respectively. The generator’s angular frequency is *ω*_*s*_. The generator’s inductance is *L*_*sd*_ and *L*_*sq*_. The permanent flux is *ψ*_*p*_, the stator resistance is *R*_*sa*_, and the number of poles is *P* [37]. Wind turbine system efficiency can be calculated as follows:

$$\eta_{WT\;system}=\frac{P_{output}\;}{P_{WT}}$$ (15)

where *P*_*W*T_ is the rated power of WT and *P*_*outpu*t_ is the output of the WT system (input power to electrolyzer). Overall efficiency for the WT system with electrolyzer is calculated according to the Eq (16):

$$\eta_{WT+Electrolyzer}=\frac{F_{H2\;}E}{P_{WT}}$$ (16)

**Fig 5:**
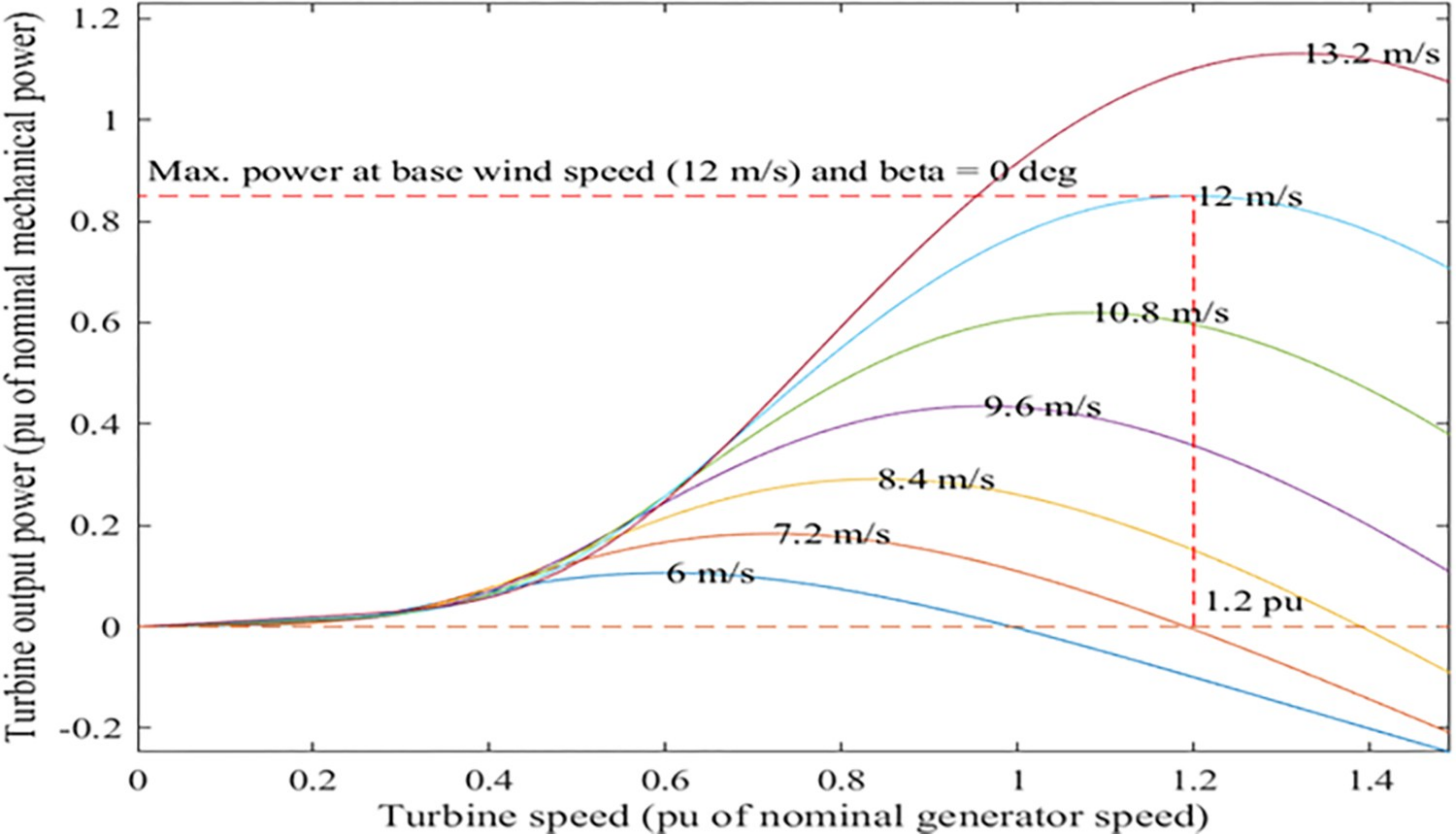
WT power characteristics at zero pitch angle.

Fig 5 displays WT properties that are applied in MATLAB [33]. The WT system parameters for simulation in MATLAB are illustrated in Table 4 [43].

**Table 4:** WT system model parameters.

**Item**	**Value**
Wind speed (fixed)	12 m/s
Pitch angle	Zero degree
Rated power	12300 W
Maximum power at base wind speed	0.85 p.u
Base rotational speed	1.2 p.u
**PMSG**	
Number of phases	3 Phases
Rotor type	Salient pole
*L*_*d*_ inductance	0.395e-3 H
*L*_*q*_ inductance	0.395e-3 H
**Rectifier**	
Type of rectifier	3-phase rectifier
Power Electronic Device	Diode
**Buck converter**	
Capacitor (*C*)	1.53e-4 F
Inductance (*L*)	4.204e-7 H
*V*_*ripple*_	2% of *V*_*out*_ (8.6 V)
Load resistance (PEM electrolyzer)	0.255 Ω
Series resistance to load	0.01 Ω
MPPT Type	P&O MPPT

### Buck converter

**Fig 6:**
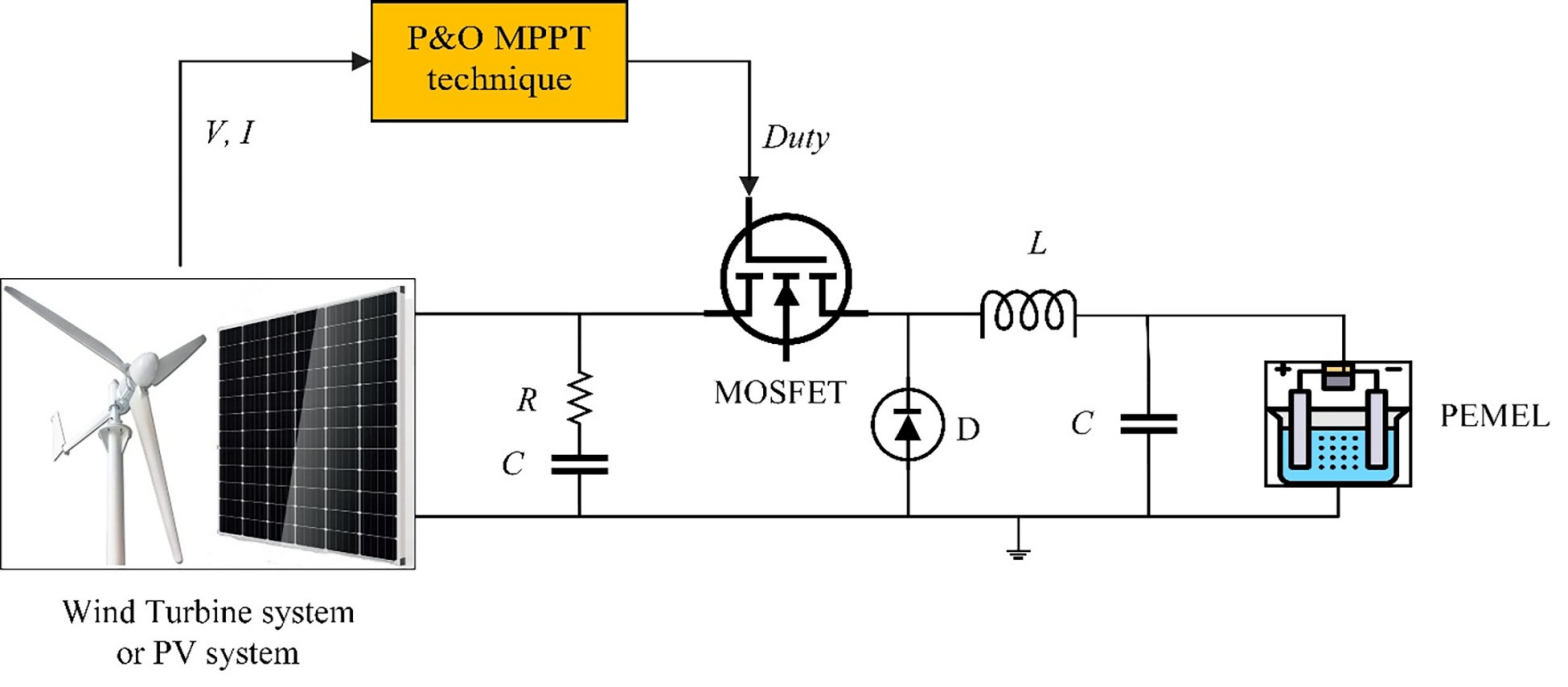
The diagram of the DC-DC buck converter.

The buck converter converts a higher dc input voltage to a lower dc output voltage. A controlled switch MOSFET, a switch diode, a capacitor (*C*), and an inductor (*L*) make up the circuit. The circuit of the buck converter is shown in Fig 6.

The capacitor ensures that current flows continuously throughout the electrolyzer by maintaining the voltage across the load and enabling currents to flow through it. The inductor is responsible for delivering and damping ripples in the load current. The capacitor and inductor work a crucial role in providing the electrolyzer with a steady current [44]. The following equations are used to compute capacitor (*C*) and inductor (*L*) [45]:

$$Duty\;cycle=\frac{V_{o}}{V_{i}}$$ (17)

$$Capacitor\;(C)=\frac{(1-D)V_{o}}{2F^{2}LV_{ripple}}$$ (18)

$$Inductor(L)=\frac{(1-D)V_{o}}{2FI_{o}}$$ (19)

Eq (20) is used to compute the PEMEL load as a resistance load:

$$Load\;resistor=\frac{V_{o}}{I_{o}}$$ (20)

### MPPT technique

**Fig 7:**
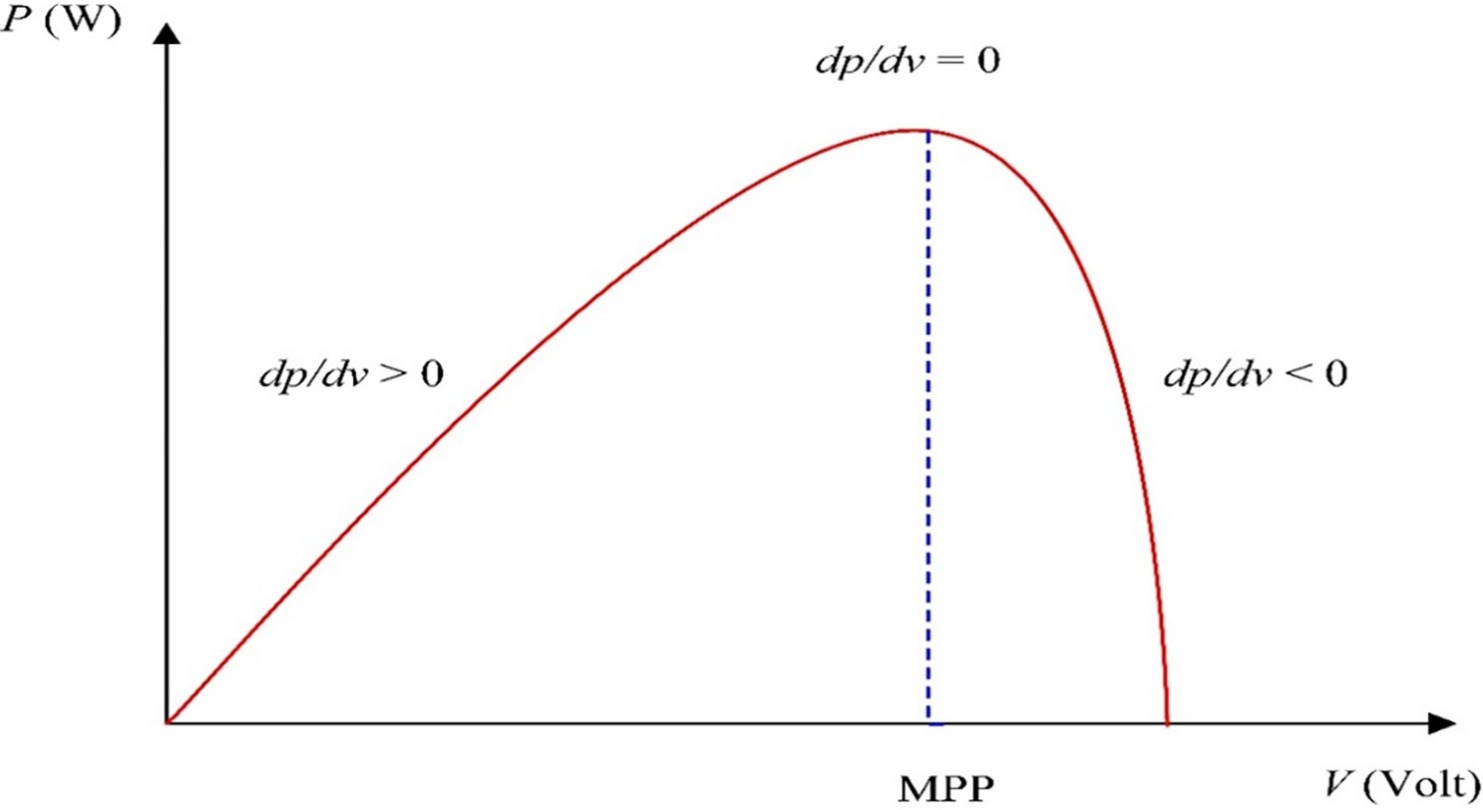
curve at various points along with the power characteristic. *P*−*V*

This model employs Perturb & Observe to gain maximum output power (MPPT). Fig 7 displays the *dP/dV* positions [38, 46, 47].

**Fig 8:**
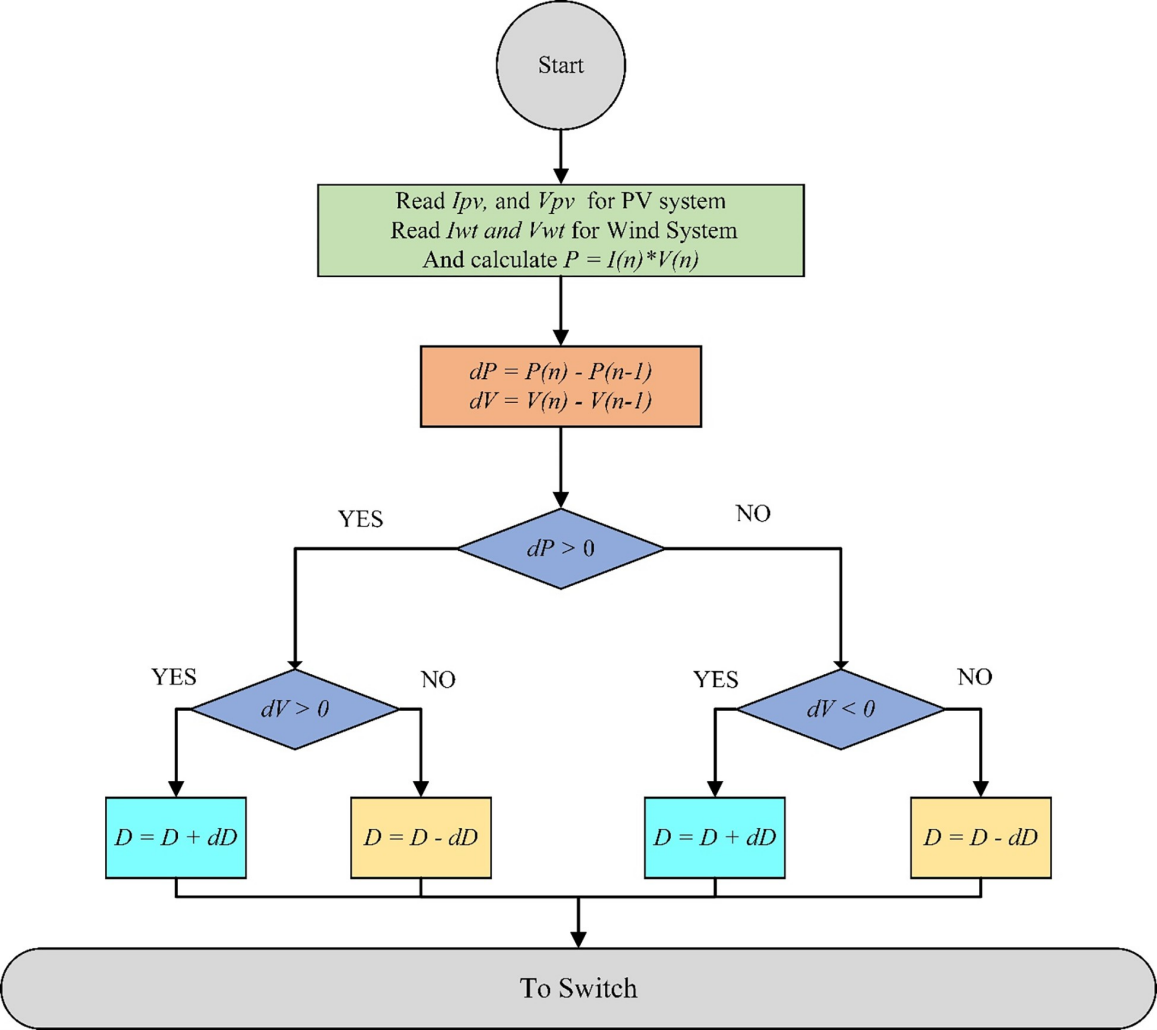
Flowchart of the P&O technique.

A little increase in this technique disrupts the PV array’s working voltage, and the resulting change in power, P, is monitored. If the P is positive, it means that the operating point has moved a bit to the MPP. This operating point should be nearest to the MPP as a result of more voltage disturbances in the same direction. If P is negative, this operating point has moved away from MPP, and the perturbation direction should then be reversed to return it to MPP [46–48]. To achieve the maximum output of power, the same method is used in wind system technology [38]. This technique is less speed than the other controllers like Fuzzy Logic Controller (FLC) and Adaptive FLC (AFLC) but we exceeded this problem by using low incremental of P&O MPPT [49, 50]. Fig 8 shows the P&O technique flowchart [31].

### MPPT code

*function D* = DutyRatio(*V*, *I*)

*D*_*max*_ = 0.95;

*D*_*min*_ = 0;

*D*_*init*_ = 0.95;

*deltaD* = 0.0001;

persistent *V*_*old*_, *P*_*old*_, and *D*_*old*_;

dataType = ’double’;

if isempty(*V*_*old*_*)*

*V*_*old*_ = 0;

*P*_*old*_ = 0;

*D*_*old*_ = *D*_*init*_;

end

P = V*I;

*dV = V-V*_*old*_;

*dP = P-P*_*old*_;

    if *dP* ~=0

      if *dP*<0

        if *dV*<0

           *D* = *D*_*old*_ + *deltaD*;

           else

           *D* = *D*_*old*_ - *deltaD*;

        end

        else

        if *dV*<0

           *D* = *D*_*old*_ - *deltaD*;

        else

        *D* = *D*_*old*_ + *deltaD*;

      end

     end

     else *D* = *D*_*old*_;

end

if *D* >= *D*_*max*_ || *D* <= *D*_*min*_

     *D* = *D*_*old*_;

end

*D*_*old*_ = *D*;

*V*_*old*_ = *V*;

*P*_*old*_ = *P*;

## Discussion of simulated results

### Investigated PV system

**Fig 9:**
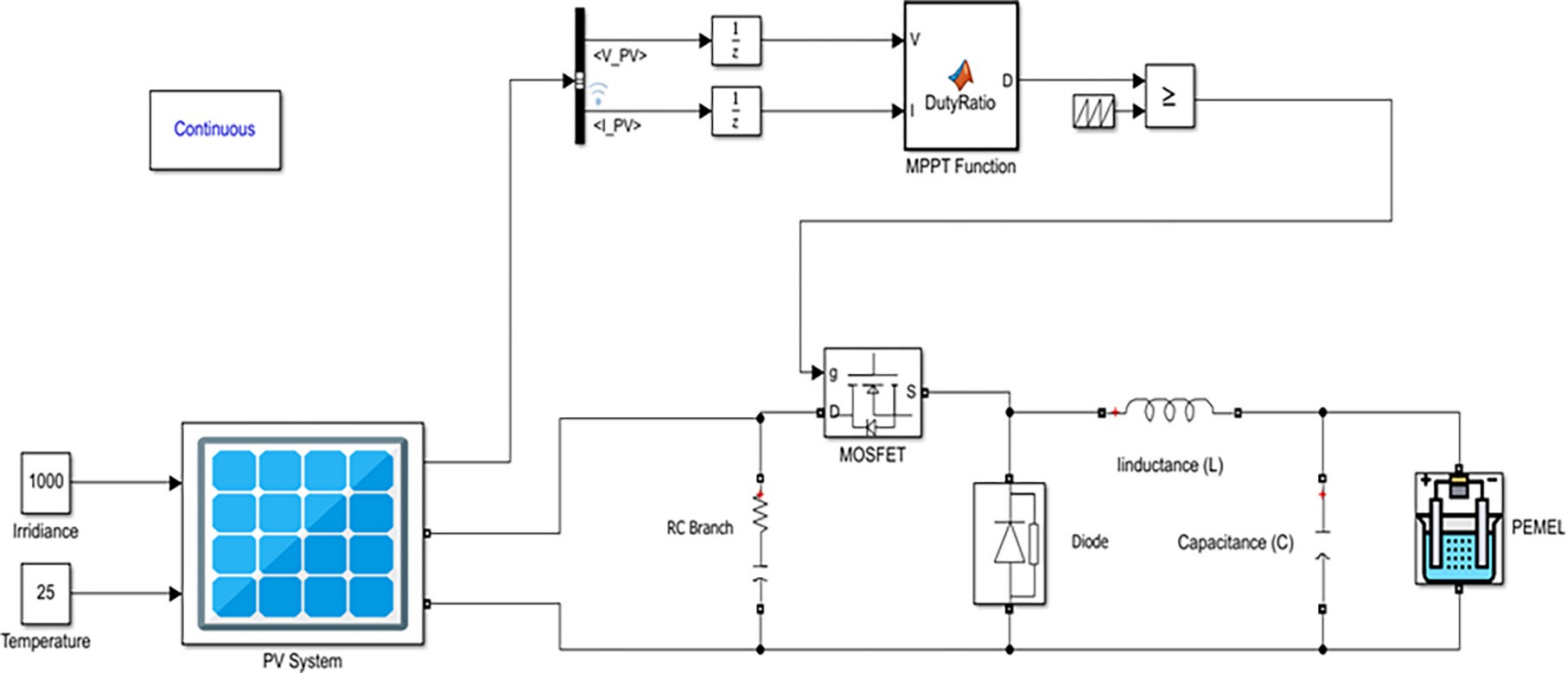
Simulation model of PV system with PEMEL in MATLAB.

A solar module, with an electrolyzer, and a buck converter circuit are combined to make a circuit to produce hydrogen. The simulation has been carried out during variable irradiance of 600, 700, 800, 900, and 1000 W/*m*^2^ and fixed temperature of 25°C. Fig 9 displays a simulation in MATLAB for PV system-powered PEMEL [33].

#### MPPT controller for PV system

Software obtained and calculated the MPPT efficiency according to input and output power for each system. All models tested under variance irradiance which was from 600 to 1000 W/m^2^ also, and the fixed temperature of 25°C.

**Fig 10:**
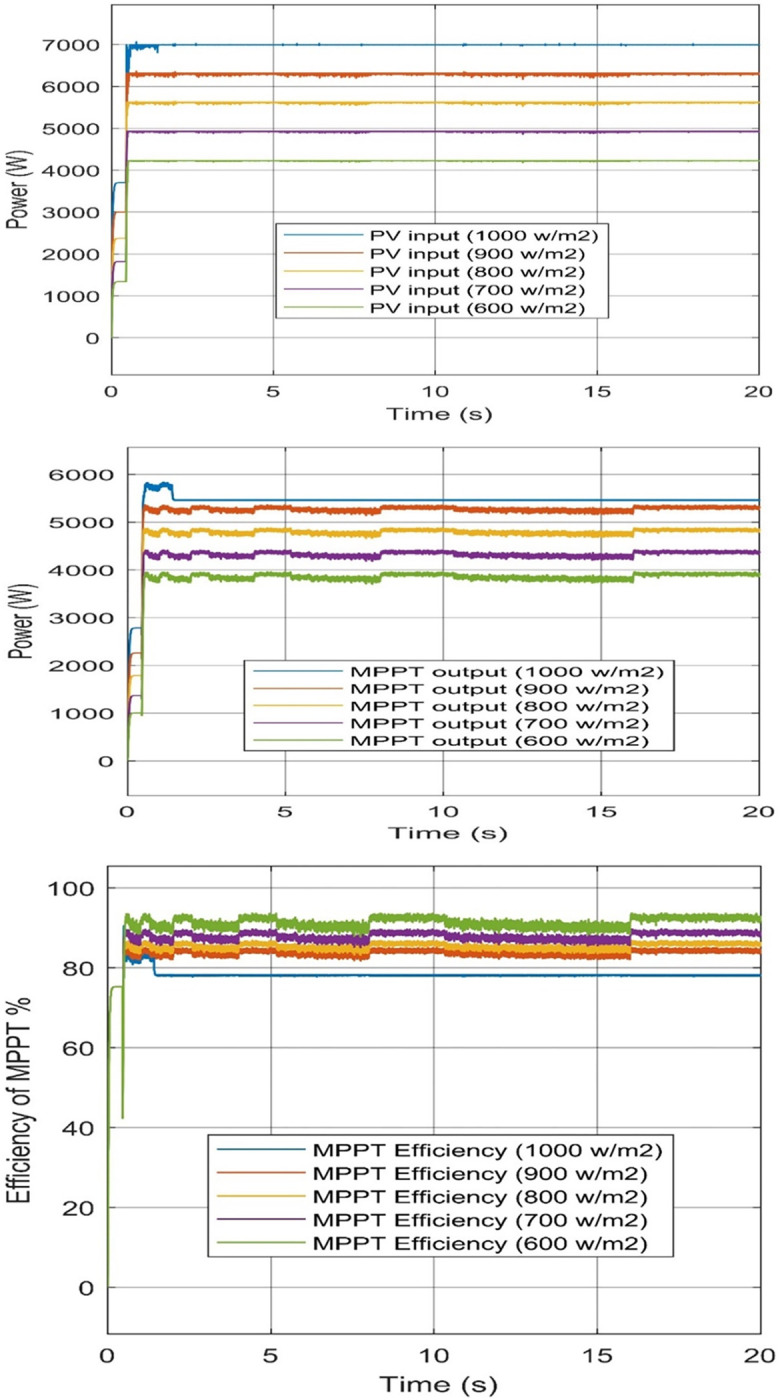
P&O-MPPT controller of PV. (a) PV inputs power, (b) controller output power, and (c) Efficiency of the controller.

The input power of the PV system to the MPPT controller at 1000 W/m^2^ equal 6993 W, at 900 W/m^2^ equal 6311 W, at 800 W/m^2^ equal 5621 W, at 700 W/m^2^ equal 4929 W, and 600 W/m^2^ equal 4228 W. The MPPT controller output at 1000 W/m^2^ equal 5472 W, at 900 W/m^2^ equal 5147 W, at 800 W/m^2^ equal 4699 W, at 700 W/m^2^ equal 4410 W, and 600 W/m^2^ equal 3961 W. Efficiency at 1000, 900, 800, 700, and 600 W/m^2^ equal 78.25%, 81.55%, 83.59%, 89.48%, and 93.69%. respectively. Fig 10 obtained input, the output of the PV system, and the efficiency of its MPPT controller.

The higher PV output power from the P&O MPPT controller, at the fixed temperature of 25°C and 1000 W/m^2^ equals 5147 W. However, the best MPPT efficiency at lower irradiance is at 600 W/m^2^ which is 93.69%. It is obtained to us from the results of MATLAB that the application of the P&O MPPT method to the PV system reaches a high efficiency for producing green hydrogen, as the results for any models were not less than 78%.

#### Voltage and current characteristics for PV system output

**Fig 11:**
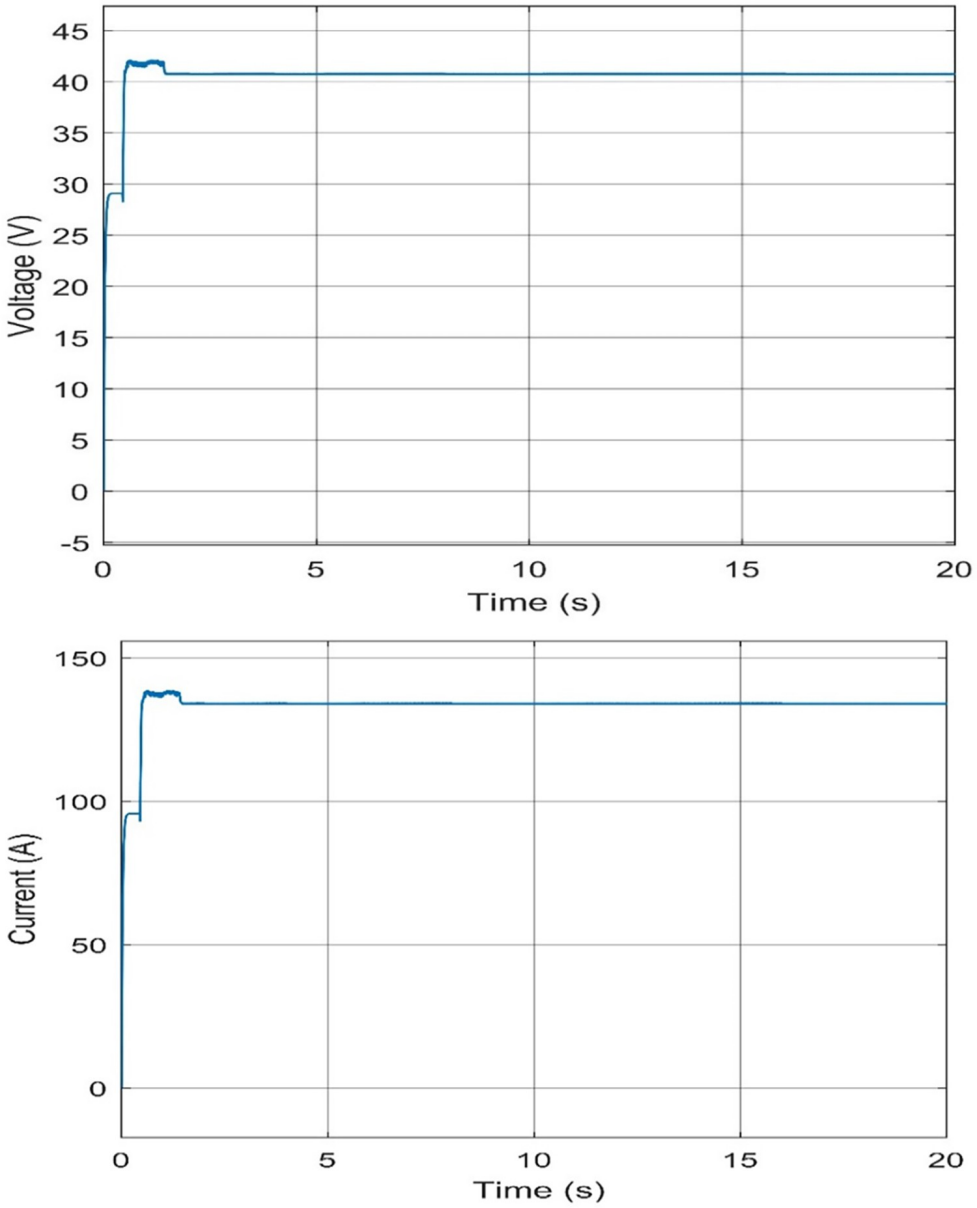
Characteristics of voltage and current output at 1000 W/m^2^ and 25°C. (a) voltage level of PEMEL, (b) current level of PEMEL.

Voltage and current characteristics are important to define the good operation of the PEMEL. From the Simulink, the operation voltage of the PEM E-130 stack is between 44 and 45 volts and the operating current for the PEM E-130 stack is 124.4 A at 1000 W/*m*^2^ and 25°C. Fig 11 shows the output voltage and current of the PV system at 1000 W/*m*^2^ and 25°C.

#### Production rate of hydrogen-powered by the PV system

**Fig 12:**
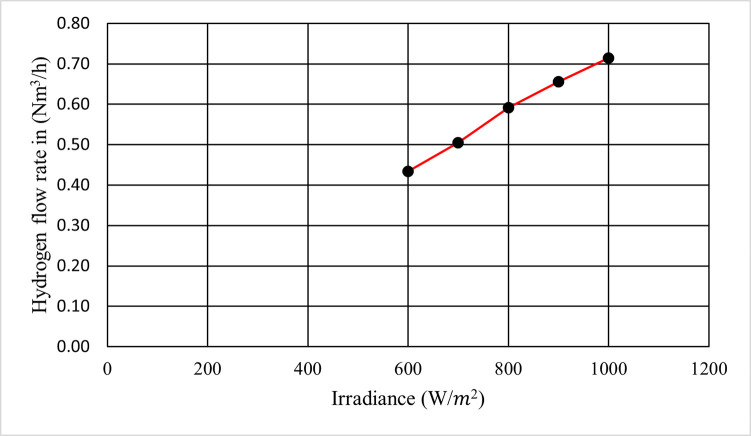
Production rate of hydrogen with variance solar irradiance.

This paper calculated Faraday’s efficiency by applying Eq (5). After that, it calculated the flow rate of hydrogen in (mol/sec) according to Eq (6). Finally, using Eq (7) to convert the hydrogen flow rate from (mol/s) to (N*m*^3^/h) is performed. The results showed the relation between irradiance in W/*m*^2^ and output hydrogen flow rate in N*m*^3^/h when the temperature was constant at 25°C as depicted in Fig 12.

### Investigated WT system

**Fig 13:**
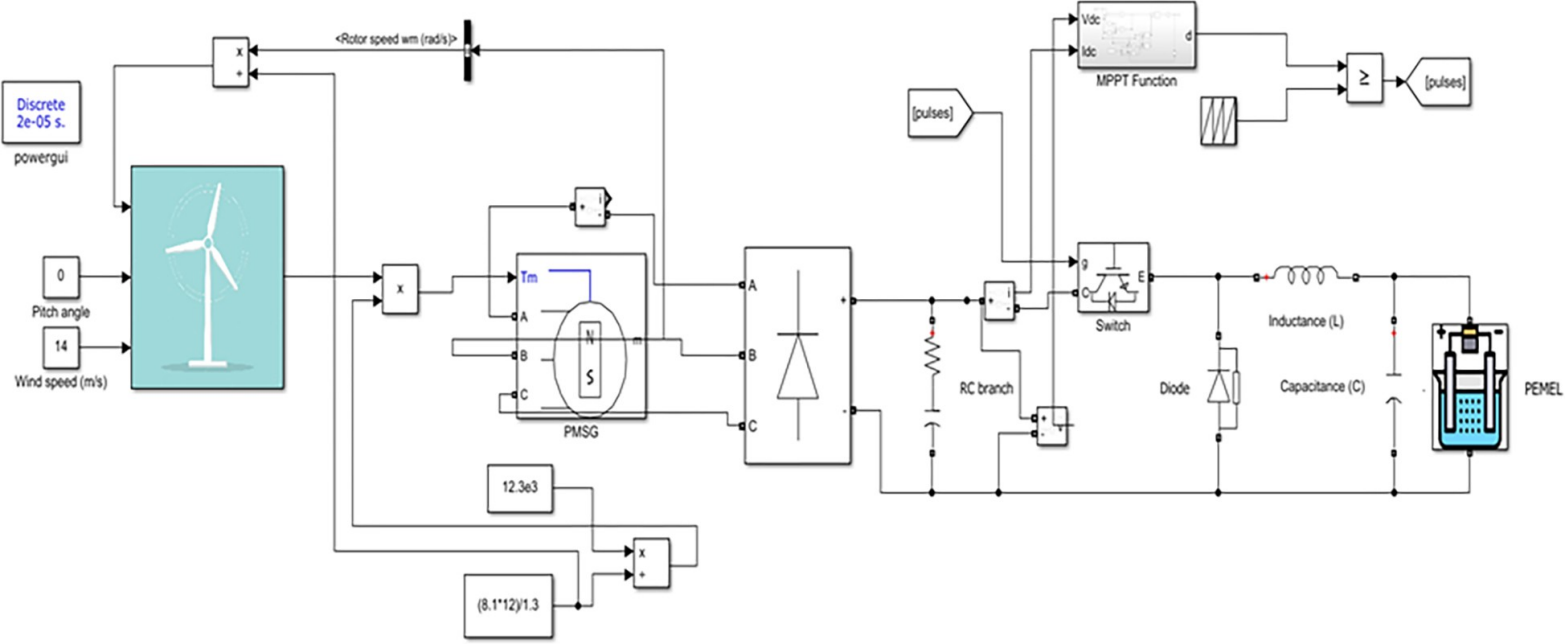
Simulation model for WT system with PEMEL in MATLAB.

The WT-powered PEMEL. MATLAB uses variable wind speed values: which are (10, 11, 12, 13, and 14 m/s) and fixed pitch angle (zero pitch angle). Fig 13 shows the simulation model of the investigated WT with PEMEL [33].

#### MPPT controller for WT system

The program calculated the MPPT efficiency according to input and output power for each system. All models tested under variance wind speed which was from W/m^2^ 14 to 10 m/s also, and the fixed pitch angle which was equal to zero.

**Fig 14:**
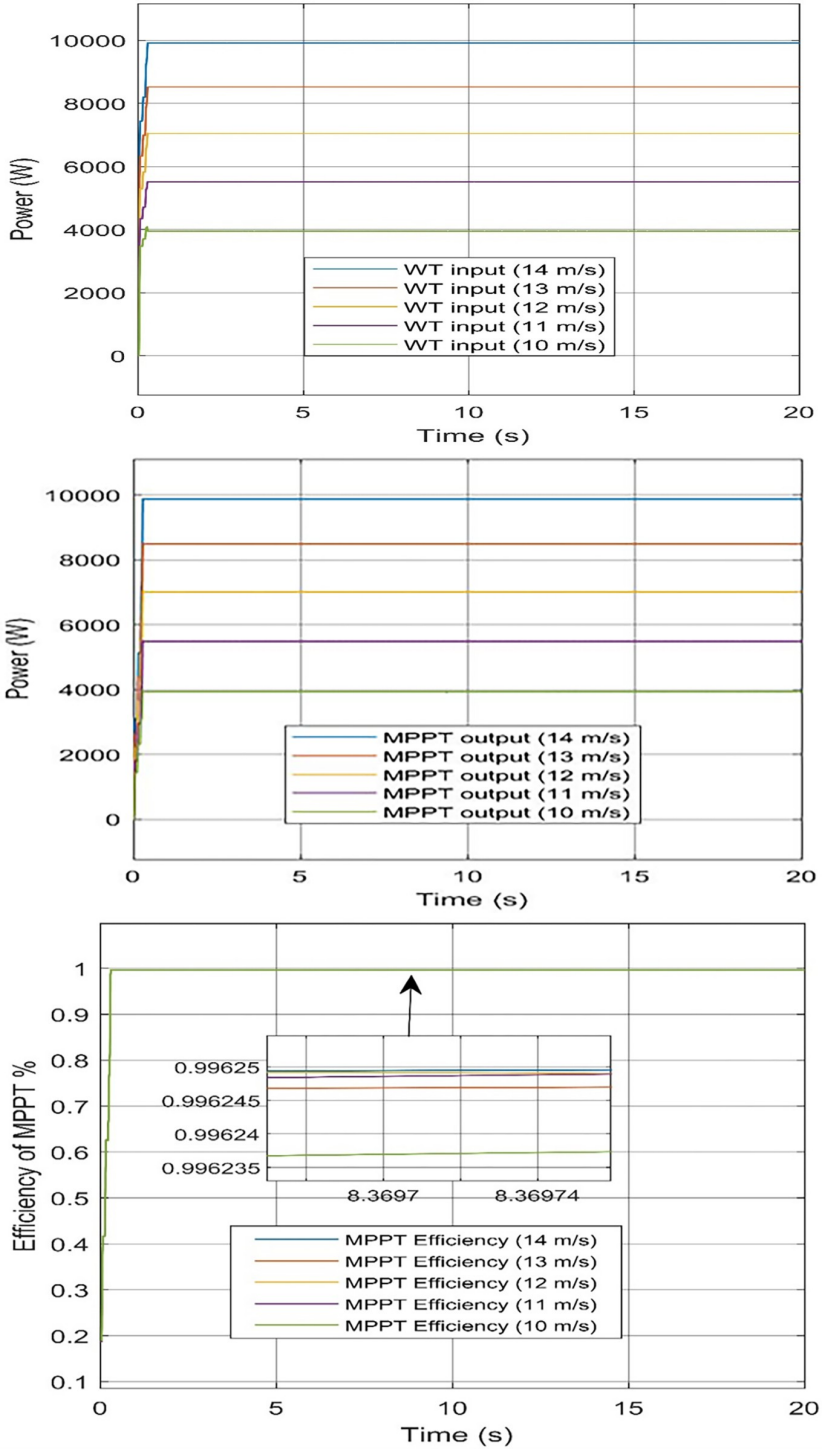
P&O-MPPT controller of WT. (a) input power to the controller, (b) output power of the controller, and (c) Efficiency of the controller.

The input power of the WT system to the MPPT controller at 14 m/s equal 9912 W, at 13 m/s equal 8521 W, at 12 m/s equal 7046 W, at 11 m/s equal 5514 W, and 10 m/s speed of wind equals 3942 W. The MPPT controller output at 14 m/s equal 9875 W, at 13 m/s equal 8489 W, at 12 m/s equal 7019 W, at 11 m/s equal 5493 W, and 10 m/s equal 3927 W. Efficiency at 14, 13, 12, 11, 10 m/s equal 99.63%, 99.62%, 99.62%, 99.62%, and 99.62%, respectively. Fig 14 obtained the input, output, and efficiency of the P&O MPPT controller.

The WT output power from the P&O-MPPT controller, at the zero fixed pitch angle and the 14 m/s speed of WT; output power equals 9912 W. Additionally, the MPPT efficiency of the different wind speeds is approximate to be fixed which equals 99.62%. It is obtained to us concluded that the implementation of the P&O MPPT algorithm to the WT system reaches not fewer than 99.62% and this is a great effect on green hydrogen technology.

**Fig 15:**
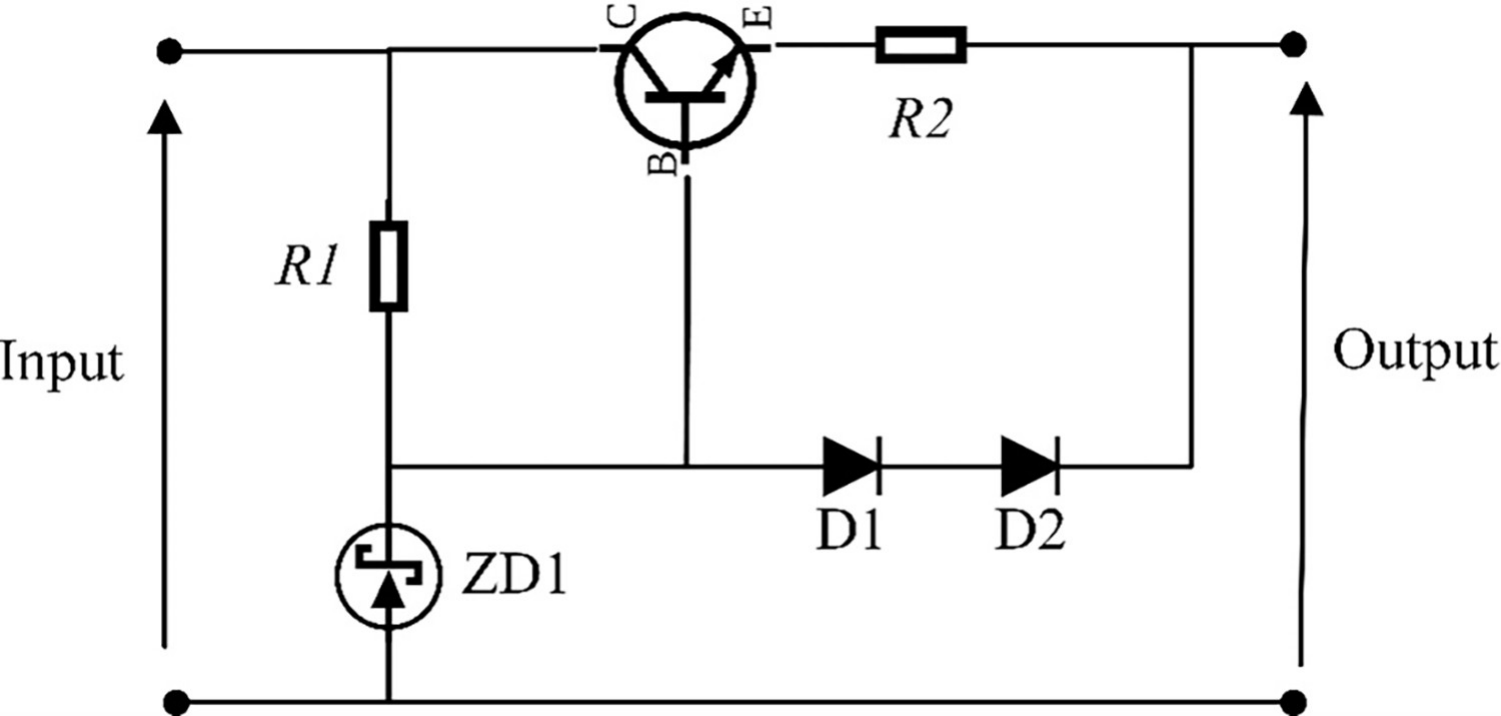
Current limiter circuit.

From the results, at 14 m/s the output power of WT is equal to 9875 W, and the current exceeded 200 A. This is not sufficient because the operating current for this PEMEL model is up to 200 A only [26]. To solve this problem, this paper suggested using the current limiter device. A sense resistor connected in series with the output pass transistor’s emitter are used in the circuit of the power supply current limiter [51]. The current is limited by two diodes that are positioned between the circuit’s output and the base of the pass transistor [51]. Fig 15 obtains the basic components of the current limiter circuit [51].

#### Voltage and current characteristics for WT system output

**Fig 16:**
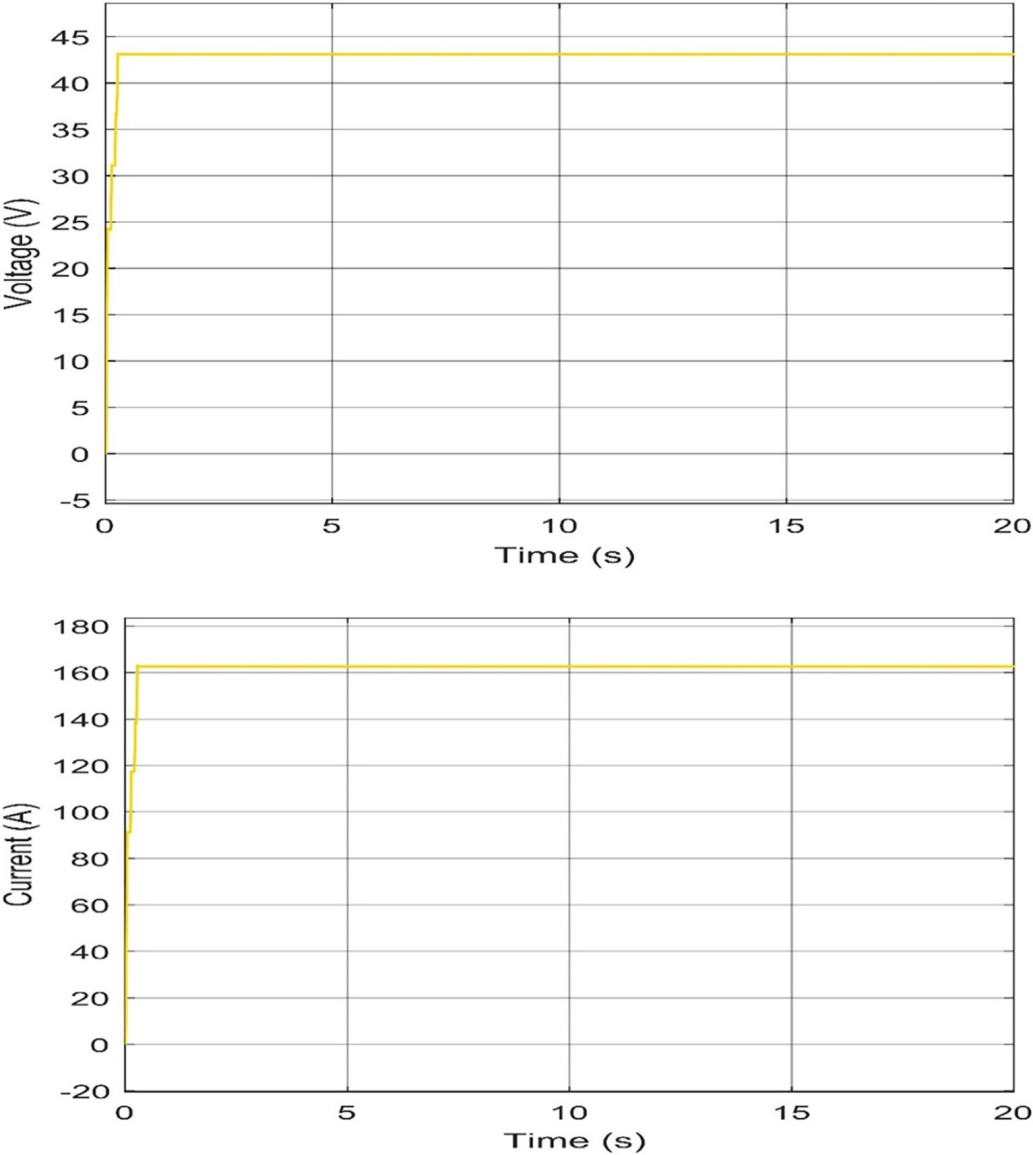
Characteristics of voltage and current output at 12 m/s and zero pitch angle: (a) Voltage Level (PEMEL), (b) Current Level (PEMEL).

The output voltage and current from WT are obtained to define the operation of the PEMEL. From the Simulink, the operation voltage of the PEM E-130 stack is between 43 and 44 Volts and the output current equals 163.2 A at 12 m/s and the zero-pitch angle. Fig 16 shows the output voltage and current of the WT system at 12 m/s and the zero-pitch angle.

#### Production rate of hydrogen-powered by WT system

This paper calculated Faraday’s efficiency using Eq (5). After that, It is calculated the flow rate of hydrogen in mol/sec according to Eq (6). Finally, It is used Eq (7) to convert the hydrogen flow rate from mol/s to N*m*^3^/h.

**Fig 17:**
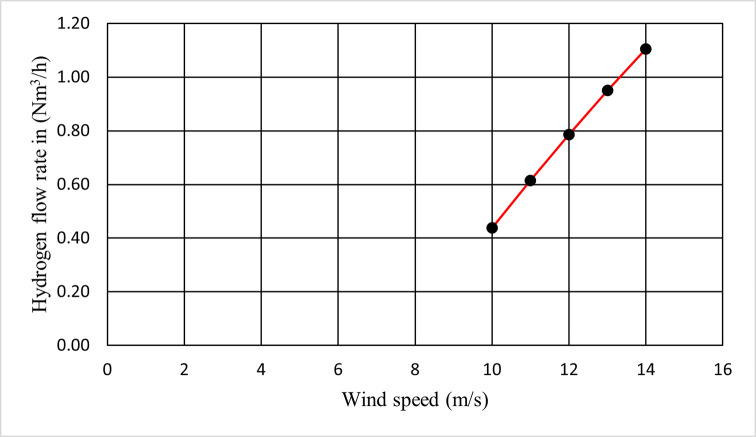
Production rate of hydrogen with variance wind speed from 10 to 14 m/s

The result is obtained in Fig 17 Which shows the relation between wind speed in m/s and the output hydrogen flow rate in N*m*^3^/h at zero pitch angle.

### Comparison between PV and WT systems to power PEMEL

The efficiency of the PV system according to Eq (9) equals 16.63%. The overall system efficiency (PV system + PEMEL) is calculated according to Eq (10) equals 5.43%.

Additionally, it calculated the efficiency of the WT system, using Eq (15), the efficiency is 57.07%. The overall system efficiency (WT system + PEMEL) using Eq (16) equals 18.77%. The comparison between the PV system and WT system-powered PEMEL is in Table 5.

**Table 5:** Comparison results between the PV system and WT system powered the PEMEL.

Type/item	Rated Power	Efficiency system	The flow rate of hydrogen (mol/s)	The flow rate of hydrogen (Nm^3^/h)	Overall system efficiency [(PV or WT system) + (PEM-E130)]
PV system at (1000 W/*m*^2^ and 25°C)	7000	16.63%	7.517e-3	0.60655	5.43%
WT system at (12 m/s and zero pitch angle)	12300	57.07%	8.073e-3	0.6514	18.77%

The results showed that the efficiency of the PV system-powered PEMEL equals 5.43% when designing a solar system with 7 kW DC power: the near-capacity of 5.7 kW of the PEMEL. The WT system powered-PEMEL efficiency is equal to 18.77% when WT with a wattage capacity of 12.3 kW: approximately to be double the capacity of the PEMEL.

The WT is more efficient than solar energy for this system selected in our study. The wind energy system exceeds three times the efficiency of the system in the case of solar energy.

#### Comparison to previous solar hydrogen systems

Other research examined the solar to hydrogen efficiency and obtained a result of 6.2% efficiency of the total system [52]. The entire effectiveness of the solar hydrogen energy unit was carefully tracked in a real-world system, and up to 5.01% overall energy efficiency [53].

Furthermore, another system has a total system efficiency at the lowest value of 1.7% [54]. In addition, a model created an optimal direct link between the electrolyzer and PV systems, leading to 9.3% of solar to hydrogen efficiency [55]. For a solar-powered PV-PEMEL, a system developed an optimization approach that raised the hydrogen generation efficiency to 12% [56].

In summary, previous solar hydrogen systems have reached solar-to-hydrogen efficiencies of less than 7% without any optimization or direct linking. This paper has up to 5.43% of its overall system efficiency: achieved when designing a PV system to the same capacity as the PEMEL.

## Conclusions

This research examines the production of hydrogen from water electrolysis using a PEMEL with solar and wind energies, which is essential in today’s world. PEMEL is more advantageous than other types of electrolyzers during to its ability to quick-starting which is important in renewable energy systems because of variances in irradiances for solar energy and wind speeds for WTs. This paper used the P&O MPPT to maximize the output power and applied it to the buck controller using low incremental steps to get the fast response of the system. This paper designed the WT for approximately double the capacity of the PEMEL using variable wind speed from 10 to 14 m/s and zero pitch angle. The solar station uses variable irradiance from 600 to 1000 W/*m*^2^ and fixed temperature which is equal to 25°C. In addition, the PV system was designed for about equivalent to the load of the PEMEL. The efficiency of wind power in extracting hydrogen is three times larger than solar energy in extracting hydrogen. This research measured the input, output, and efficiency of the P&O MPPT system. As well as this paper showed the characteristics of voltage and current for each system. In addition, this research made a comparison between PV and WT which powered the PEMEL separately. According to the findings, almost two times as much WT electricity should be used to feed PEMEL, and a PV system is as same as the capacity of PEMEL. This paper can be a reference for designing PV or WT to feed a PEMEL in practical life.

In our opinion, future work will focus in:

- Adding one of the energy storage solutions to solve the problem of changing output power and characteristics as a result of variation in solar irradiance and wind speed like the BESS and supercapacitors.
- Development of MPPT function and making the comparison between P&O, FLC, and Adaptive FLC to show the best method to get maximum power that can be extracted from any renewable energy source.
- Increasing the efficiency of solar panels so that they can produce more power in the same area as the solar panels currently.
- Improvement and analysis in the case that the electrical network is connected to a green hydrogen system that received power from a renewable energy source.
- Increasing the stability of power electronic circuits by optimizing the design of systems.

## Supporting information

S1 File (RAR)

## Abbreviations

λ: Tip speed ratio
η_*F*_: Faraday efficiency
C_p_: The power coefficient of wind turbine
*F*_*H*2_: Electrolyzer hydrogen flow rate (mol/s
C: Capacitor
°C: Degrees Celsius
D: Duty cycle
F: Farad
H: Henry
L: Inductor
N: Electrolyzer cell number
n: Number of electron exchanges
P: Power
p.u: Per unit
β: The blade pitch angle
ρ: The air density
AEL: Alkaline electrolyzer
BESS: Battery energy storage systems
HHV: Higher heating value
HRES: Hybrid renewable energy system
IRENA: International renewable energy agency
KPI: Key performance indicators
LCOE: Levelized cost of electricity
MBA: Mine blast algorithm
MOSFET: The metal–oxide–semiconductor field-effect transistor
MPPT: Maximum power point tracking
NREL: National renewable energy laboratory
NSGA-II: Non-dominated sorting genetic II
P&O: Perturb & observe
PEM: Polymer electrolyte membrane
PEMEL: Polymer electrolyte membrane electrolyzer
PMS: Power management system
PMSG: Permanent magnet synchronous generator
PSO: Swarm optimization
PV: Photovoltaics
SPE: Solid polymer electrolyte
ST: Storage tank
WT: Wind turbine
